# Is too ‘creative’ language acceptable in crystallography?

**DOI:** 10.1107/S090744491002799X

**Published:** 2010-08-13

**Authors:** Alexander Wlodawer, Jacek Lubkowski, Wladek Minor, Mariusz Jaskolski

**Affiliations:** a Macromolecular Crystallography Laboratory, NCI, Frederick, MD, USA; b University of Virginia, Charlottesville, VA, USA; c A. Mickiewicz University, Poznan, Poland

**Keywords:** letters to the editor, crystallographic terminology

## Abstract

While figures of speech are often useful and even educational, flashy titles combined with hyperbolae and imprecise language can mislead or deceive non-specialist readers and should therefore be avoided. The possibility of such confusion exists when poorly defined terms like ‘structure quality’ or ‘super-resolution’ are used to describe a protein structure.

## Abstract 2

A brief comment is made on the need to use carefully selected, novel terms in crystallographic publications, especially publications addressing non-specialists.

One reason why X-ray crystallography has been so successful for the almost 100 years of its history is that its language is very precise. Such terms as ‘resolution’ or ‘*R* factor’ are uniquely defined, at least to the readers of this journal. On the other hand, such plain and common-sense terms as ‘data quality’ and ‘structure quality’, often used with reference to the results of crystallographic research, are actually not very precise and may have a different meaning in different situations, especially in macromolecular crystallography. One could reasonably postulate that the highest ‘structure quality’ would represent the lowest deviation from the true structure (thus highest accuracy), but since such information is not known for novel structures (as opposed to test cases), other less direct indicators, such as free *R* or deviation from acceptable geometry, have to be used instead.

The language of our discipline is certainly not set in stone and, indeed, absorbs many novel terms, or innovative combinations of existing phrases. However, the new terminology should be selected judiciously to avoid ambiguity and/or misleading meaning. In particular, authors of crystallographic papers, especially published in high-impact general-interest journals, should make a clear distinction between structure ‘resolution’ and ‘quality’ in order not to confuse readers not versed in crystallographic terminology. For example, this linguistic Puritanism has not been observed in a recent article discussing the use of deformable elastic networks (DEN) in macromolecular refinement, which has a rather surprising title (*Super-resolution biomolecular crystallography with low-resolution data*) (Schröder *et al.*, 2010 ▶). Its authors propose, quite controversially in our understanding of the terms, that

X-ray structures can achieve ‘super-resolution’, where the estimated coordinate accuracy is better than the resolution limit of the diffraction data (typically by 10 times), by imposing constraints when interpreting observed diffraction data and electron density maps

or that

a structure derived from low-resolution diffraction data can have quality similar to a high-resolution structure.

We want to stress that we have no intention to contest the results of Schröder *et al.* which show that, by analogy to the use of geometrical restraints, refinement of low-resolution structures that includes DEN restraints might improve the convergence of the method and the quality of the final structures. We are quite convinced that the DEN method will be helpful for improving the quality of the structures of biological complexes, but, as stated above, we argue that the term ‘quality’ is by no means synonymous with ‘resolution’, and that these two aspects of published structures should not be confused. A similar subject had been previously discussed in the context of the claims that the resolution obtained in optical microscopy could exceed the Abbe’s limit (Stelzer, 2002 ▶), with the conclusion that whereas the quality of the images was improved by using a clever computational technique, the resolution was not. We suspect that the use of the disputed terms in the paper by Schröder *et al.* represents a similar case.

An indication that low-resolution structures are not as accurate as their high-resolution counterpart can actually be found in the paper by Schröder *et al.* The authors tested their method by refining penicillopepsin with synthetic low-resolution data, starting from a homology model derived from endothiapepsin. The resulting coordinates deviate from the target by as much as 1.5 Å (see Fig. 1 of Schröder* et al.*) which is hardly comparable to the error of ~0.2 Å that can be estimated for the structure refined with the original data extending to 1.8 Å resolution. It is our opinion that the only cases in which low-resolution structures could match the quality of their high-resolution counterparts would be when the latter were incorrectly solved or refined – a situation very rare among the more than 56 000 crystal structures currently deposited in the Protein Data Bank, and almost absent at truly high resolution.

It has been generally known since the beginning of crystallographic research that the accuracy of the coordinates of individual atoms is much higher than the resolution of the data, even in the absence of restraints. For example, small-molecule structures are usually refined at resolution not exceeding 0.7 Å, but the atomic positions are often accurate to ∼0.001 Å. It has also been known for over 40 years that in the absence of restraints and/or constraints it is not possible to refine protein structures at lower than atomic resolution. Also, the fact that seems to be the main source of confusion in the paper by Schröder *et al.*, namely that the coordinates resulting from restrained/constrained refinement have much lower deviation from the target values than the nominal resolution of the data, has been known for a long time. Schröder *et al.* refer to the work of Luzzati (1952 ▶), but it is surprising that they have not used the diffraction-component precision index (DPI) introduced by Cruickshank (1999 ▶) to benchmark their method. The DPI index is the best tool we have to estimate the quality of macromolecular models of different resolutions, refined under restraint control.

Thus, we do not believe that the phrase ‘super-resolution crystallography with low-resolution data’ is a very fortunate one. We feel that as authors of crystallographic papers, especially those published in non-specialist journals, we should be very careful in the use of terms and statements if they could be misinterpreted. With the lack of crystallographic education among young scientists and black-box approach to structure solution and refinement, careful use of termin­ology is essential. While figures of speech are often useful, flashy titles combined with hyperbolae and imprecise language can mislead or deceive non-specialist readers and should be therefore avoided.
